# Remote tropical island colonization does not preclude symbiotic specialists: new evidence of mycorrhizal specificity across the geographic distribution of the Hawaiian endemic orchid *Anoectochilus sandvicensis*

**DOI:** 10.1093/aob/mcy198

**Published:** 2018-10-31

**Authors:** Sean Swift, Sherilyn Munroe, Chaewon Im, Laura Tipton, Nicole A Hynson

**Affiliations:** 1 Department of Botany, University of Hawaii Manoa, Honolulu, HI, USA; 2 John A. Burns School of Medicine, University of Hawaii Manoa, Honolulu, HI, USA; 3 Pacific Biosciences Research Center, University of Hawaii Manoa, Honolulu, HI, USA

**Keywords:** *Anoectochilus sandvicensis*, *Ceratobasidium*, island biogeography, mycorrhizal fungi, Orchidaceae, rhizoctonia, tropical ecology

## Abstract

**Background and Aims:**

For symbiotic organisms, their colonization and spread across remote oceanic islands should favour generalists. Plants that form obligate symbiotic associations with microbes dominate island ecosystems, but the relationship between island inhabitance and symbiotic specificity is unclear, especially in the tropics. To fill this gap, we examined the mycorrhizal specificity of the Hawaiian endemic orchid *Anoectochilus sandvicensis* across multiple populations encompassing its entire geographic distribution.

**Methods:**

By molecular phylogenetic approaches we identified the mycorrhizal fungi associated with *A. sandvicensis* across its entire geographic distribution and determined the relationship of these fungi to others found elsewhere around the globe. With richness estimators, we assessed the mycorrhizal specificity of *A. sandvicensis* within and among islands. We then tested whether geographic proximity of orchid populations was a significant predictor for the presence of particular mycorrhizal fungi and their community composition.

**Key Results:**

We found that each population of *A. sandvicensis* forms specific associations with one of three fungi in the genus *Ceratobasidium* and that the closest relatives of these fungi are globally widespread. Based on diversity indices, *A. sandvicensis* populations were estimated to partner with one to four mycorrhizal taxa with an estimated total of four compatible mycorrhizal fungi across its entire distribution. However, the geographic proximity of orchid populations was not a significant predictor of mycorrhizal fungal community composition.

**Conclusions:**

Our findings indicate that the colonization and survival of plant species on even the most remote oceanic islands is not restricted to symbiotic generalists, and that partnering with few, but cosmopolitan microbial symbionts is an alternative means for successful island establishment. We suggest that the spatial distribution and abundance of symbionts in addition to island age, size and isolation should also be taken into consideration for predictions of island biodiversity.

## INTRODUCTION

Carlquist in his classic work *Island biology* (1974) posited that the successful colonization of islands by obligate symbiotic organisms such as insect-pollinated plants, would favour generalists. Recent studies of plant–pollinator networks on islands provide some empirical support for Carlquist’s hypothesis, where native and endemic plants along with their pollinators tend to be generalists and less specific than expected by chance (Castro-Urgal and Traveset, 2014; Trojelsgaard *et al.*, 2015; Hervias-Parejo and Traveset, 2018). However, the degree of symbiont specificity across the geographic distributions of island native, and especially endemic, host species remains unknown for many organisms such as mycorrhizal plants. Here, we examine the interaction between symbiotic specificity and island colonization across the entire distribution of a species of a terrestrial orchid that is endemic to the main Hawaiian Islands.

Orchids provide an ideal study system to examine the interactions between island inhabitation and symbiont specificity. Family Orchidaceae is one of the largest on earth, with species existing on hundreds of islands and every continent except Antarctica (Dressler, 2005). Also, orchids require fungal symbionts in their earliest stages of development due to their production of dust seeds that lack endosperm (Rasmussen, 1995). These mycorrhizal fungi colonize orchid seedlings and provide all necessary carbohydrates and nutrients to the developing embryo, and often remain associated with the orchid when it becomes autotrophic (Smith and Read, 2008). The degree to which adult orchids ‘repay’ their mycorrhizal symbionts with photosynthetically derived carbon remains unclear (Cameron *et al.*, 2008), and the specificity of orchid–fungal associations can shift from specific to more general over the course of orchid ontogeny (Bonnardeaux *et al.*, 2007; Bidartondo and Read, 2008). The dominant fungi that form mycorrhizal associations with orchids are in the polyphyletic group known as the rhizoctonias, which includes species in the families Tulasnellaceae, Ceratobasidiaceae and Serendipitaceae (Dearnaley *et al.*, 2016; Weiß *et al.*, 2016). Unlike other lineages of mycorrhizal fungi, some orchid-associated species are thought to be capable of living independently of a host as soil saprotrophs (Veldre *et al.*, 2013; Weiß *et al.*, 2016), indicating that their geographic distributions may not be strictly tied to those of their hosts (McCormick and Jacquemyn, 2014). Based on a recent biogeographic report, orchid-associated Ceratobasidiaceae and Tulasnellaceae appear to be globally widespread, while Serendipitaceae species are less common (Jacquemyn *et al.*, 2017). However, distribution data on orchid mycorrhizal fungi are patchy, especially for the tropics where some of the greatest orchid diversity occurs (Swarts and Dixon, 2009).

Symbiont diversity in orchids can range from highly specific associations with single species of fungi, to much more general interactions with multiple fungal lineages (Shefferson *et al.*, 2005; McCormick *et al.*, 2006; Dearnaley, 2007; Waterman and Bidartondo, 2008; Jacquemyn *et al.*, 2010). While information on the mycorrhizal associations of island-inhabiting orchids is limited, previous studies provide mixed support for Carlquist’s predictions. Some studies have found high degrees of mycorrhizal specificity among native island orchids, while others have found them to be generalists associating with a diversity of rhizoctonias, but favouring Tulasnellaceae species (Otero *et al.*, 2002; Ma *et al.*, 2003; Kristiansen *et al.*, 2004; Otero *et al.*, 2004; Martos *et al.*, 2012; Jacquemyn *et al.*, 2017). Information is lacking particularly on the mycorrhizal specificity and associations of tropical island orchids, many of which are threatened or endangered with extinction (Swarts and Dixon, 2009). Furthermore, previous studies are limited in their ability to examine explicitly the relationship between island inhabitance by orchids and mycorrhizal specificity due to: (1) many of the orchid species examined thus far have distributions that include islands and continents; and (2) when mycorrhizal specificity has been detected in island endemic orchids, these observations are limited to one or two orchid populations. Here we leverage the presence of an island orchid *Anoectochilus sandvicensis* which occupies six discrete oceanic islands to examine the degree of symbiont specificity among multiple host populations and across the host’s entire geographic distribution.

The Hawaiian Islands provide a unique backdrop for biogeographic studies. Hawaiʻi is the most isolated archipelago on the planet, made up of eight main islands that range in age and size from 0.4 to 5.1 million years old and from 115.5 to 10 432 km^2^, respectively (Ziegler, 2002). From Niʻihau, the oldest and furthest west of the main Hawaiian Islands, to the youngest and furthest east Island of Hawaiʻi spans approx. 620 km, while only 11 km separate the closest main islands of Maui and Kahoʻolave; each island also harbours unique biomes ranging from tropical rain forest to volcanic deserts. Accordingly, the species that have arrived there have done so by improbable means and many have undergone rapid radiations (Ziegler, 2002). This has resulted in unique flora and fauna, with an estimated 89 % of Hawaiʻi’s vascular plant species found nowhere else in the world (Wagner *et al.*, 1999). Despite their propensity for long-distance dispersal (Givnish *et al.*, 2016), Hawaiʻi harbours only three distantly related native orchids, including *A. sandvicensis* (tribe Cranichideae) (Wagner *et al.*, 1999).

Due to Hawaiʻi’s extreme isolation and the requirement of all orchids for symbiotic mycorrhizal fungi, we predicted that the orchids that inhabit these islands should be mycorrhizal generalists (*sensu*Carlquist, 1974). Specifically, we set out to test the hypotheses that: (1) *A. sandvicensis* partners with a diversity of mycorrhizal fungal lineages across its geographic distribution; and (2) as distance among *A. sandvicensis* populations increases, the similarity of mycorrhizal fungal communities decreases.

The fact that *A. sandvicensis* is endemic and only found on the main Hawaiian Islands makes it an interesting study system to examine the interactions between endemism and symbiont specificity. Due to the isolation of Hawaiʻi, one might assume that endemic species that require mutualists may, by default, be specialists due to a limited number of available and compatible symbionts. However, this appears not to be the case for island endemic plants and their pollinators (Castro-Urgal and Traveset, 2014; Trojelsgaard *et al.*, 2015; Hervias-Parejo and Traveset, 2018). While there is conflicting information on the degree of mycorrhizal specificity among endemic vs. more widespread orchids (Swarts *et al.*, 2010; Phillips *et al.*, 2011; Jacquemyn *et al.*, 2016) based on evidence from relatives of *A. sandvicensis*, there is some support that this genus is capable of forming mycorrhizae with a diversity of rhizoctonia fungi, and even one normally saprotrophic species of *Mycena* (Guo *et al.*, 1997; Jiang *et al.*, 2015). Of the three endemic Hawaiian orchids, we chose to focus on *A. sandvicensis* because, while locally rare, it is the most common native orchid species in Hawaiʻi found throughout all the major islands including Maui, Oʻahu, Hawaiʻi, Kauaʻi, Molokaʻi and Lānaʻi, and its habitats range from montane wet tropical forests to mesic shrublands. We used a molecular and phylogenetic approach to determine the identities and evolutionary relatedness of the mycorrhizal fungi associated with 12 populations of *A. sandvicensis* from four islands representing the full extent of its geographic distribution. Using richness estimators, we assessed the mycorrhizal specificity of *A. sandvicensis* among populations and across its distribution, and then tested the relationship between geographic distance among orchid populations and mycorrhizal fungal community similarity.

## MATERIALS AND METHODS

### Field sites and sampling

With the assistance of local botanists, populations or clusters of *Anoectochilus sandvicensis* were located on the islands of Oʻahu, Maui, Kauaʻi and Hawaiʻi (Table 1). Once research permits were obtained for each site, between July 2013 and April 2017 we sampled roots from 12 *A. sandvicensis* populations (AN1–AN12) across the four islands (three on Oʻahu, four on Maui, three or four on Kauaʻi and one on Hawaiʻi Island, Table 1). In general, populations were separated by >100 m, except for those found on the steep and difficult to access Kahili Ridge, Kauaʻi where the two closest (AN8 and AN10) were 1.8 m apart, making it challenging to confirm *in situ* that they were separate populations. Measured using the GPS coordinates of our sampling sites, the greatest distance between populations was approx. 532 km between AN11 from Wainiha Valley on Kauaʻi and AN12 from the Olaʻa tract in Volcanoes National Park on Hawaiʻi Island (Table 1). Vegetation types for orchid collection sites were lowland to montane, wet to mesic, native forests and shrublands.

**Table 1.** Orchid population site locations for samples collected in this study and fungal operational taxonomic units (OTUs) identified from each population including new accession numbers and best BLAST match from NCBI’s GenBank, and number of representative sequences per OTU

Location	Population	Elevation (m)	Fungi identified	Accession number	Best BLAST match name and accession number	No. of representative sequences	Maximum score	Query coverage (%)	Per cent identity (%)
Mt. Ka’ala, Oʻahu	AN01	1208	*Ceratobasidium* sp. 1	MF539823	*Ceratobasidium* sp. AB290022.1	21	1046	100	98
			Tulasnellaceae sp.		*Tulasnella* sp. JN655638.1	2	1119	98	90
			Polyporales sp. 1		*Polyporous tricholoma* KP943502.1	1	398	83	81
			Polyporaceae sp.		*Coriolopsis rigida* JF894112.1	1	1079	93	99
	AN02	1195	*Ceratobasidium* sp. 2	MF539822	Ceratobasidiaceae sp. JX138539.1	9	1138	100	98
	AN03	1227	*Ceratobasidium* sp. 2	MF539822	Ceratobasidiaceae sp. JX138539.1	19	1138	100	98
			Polyporales sp.		*Polyporous tricholoma* KP943502.1	2	398	83	81
Waikamoi Nature Preserve, Maui	AN04	1392	*Ceratobasidium* sp. 1	MF539823	*Ceratobasidium* sp. AB290022.1	5	1046	100	98
			Trechisporales sp.		*Subulicystidium perlongisporum* KP268489.1	4	327	99	85
			*Galerina* sp.		*Galerina* sp. JX029941.1	1	1050	100	94
	AN05	1447	*Ceratobasidium* sp. 1	MF539823	*Ceratobasidium* sp. AB290022.1	30	1046	100	98
			*Galerina* sp.		*Galerina* sp. JX029941.1	1	1046	100	98
	AN06	1476	*Ceratobasidium* sp. 1	MF539823	*Ceratobasidium* sp. AB290022.1	26	1046	100	98
	AN07	1391	*Ceratobasidium* sp. 1	MF539823	*Ceratobasidium* sp. AB290022.1	21	1046	100	98
Kahili Ridge, Kauaʻi	AN08	829	*Ceratobasidium* sp. 1	MF539823	*Ceratobasidium* sp. AB290022.1	7	1046	100	98
	AN09	810	*Ceratobasidium* sp. 1	MF539823	*Ceratobasidium* sp. AB290022.1	12	1046	100	98
		810	Atheliaceae sp.		*Atheliaceae* sp. JX898945.1	3	1046	100	98
	AN10	830	*Ceratobasidium* sp. 1	MF539823	*Ceratobasidium* sp. AB290022.1	19	1046	100	98
Wainiha Valley, Kauaʻi	AN11	823	*Ceratobasidium* sp. 3	MF539824	*Rhizoctonia* sp. JQ859895.1	18	924	100	96
Ola’a tract, Volcano National Park, Hawaiʻi	AN12	1162	*Ceratobasidium* sp. 2	MF539822	Ceratobasidiaceae sp. JX138539.1	35	1138	100	98

Using a trowel cleaned with 70 % ethanol, we excavated small rhizome and root sections from each population of *A. sandvicensis*. Due to the rhizomatous and mat-forming growth habit of *A. sandvicensis*, we were unable to determine individuals in the field, so root samples are representative of clusters of intertwined individuals no more than 0.5 m apart. Root samples between 3 and 6 cm long were placed into plastic bags and put directly on ice. After returning from the field, samples were washed with tap water and sectioned with clean razor blades into small sub-samples approx. 1–2 cm long that were stored in 600 μL of cetyltrimethylammonium bromide (CTAB) buffer. These samples were frozen at 0 ºC until they were returned to the University of Hawaiʻi at Mānoa where they were stored at –20 °C for future molecular analyses.

### Root sectioning

**Fig. 1.**
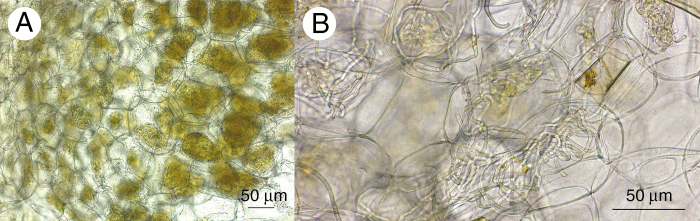
Micrographs of root cross-sections from *Anoectochilus sandvicensis*, (A) ×20 magnification of fungal pelotons within orchid root cells, (B) ×40 detail of fungal hyphae that make up the pelotons within the root cells of *A. sandvicensis*.

Replicate root samples of *A. sandvicensis* from each population were removed from CTAB buffer, surface sterilized in a 10 % bleach solution for 30 s and suspended in water immediately prior to sectioning. Each root sample was sub-divided into 0.5 mm transverse sections. For each root sub-sample, 3–4 sections that showed signs of colonization (dense fungal pelotons in cortical cells, Fig. 1) were selected for DNA isolation. Colonized sections from each root sub-sample were placed in 2 mL bead beating tubes with 1000 μL of CTAB and sterile 2 mm and 5 mm glass beads, and stored at –20 °C prior to DNA isolation. We extracted DNA from a minimum of 16 samples per orchid population.

### Molecular methods

Colonized root sections suspended in CTAB buffer were thawed at 65 °C and frozen in liquid nitrogen three times to soften tissue prior to bead homogenization. Samples were homogenized in a mini beadbeater (Biospec) and then incubated at 65 °C for 1 h and mixed by inverting every 20 min. DNA was extracted using the Qiagen DNeasy Blood & Tissue kit (Qiagen Inc., Valencia, CA, USA) following a modified protocol (Hynson and Bruns, 2009). Using PCR, the nuclear ribosomal internal transcribed spacer (ITS) region was amplified with the orchid mycorrhizae optimized primer combination ITS1OF/ITS4OF and, for a sub-set of samples, the primer combination ITS1/ITS4-TUL and the PCR conditions described in Taylor and McCormick (2008). For representatives of each *Ceratobasidium* operational taxonomic unit (OTU; see the Results), a larger fragment of the ribosomal large sub-unit (LSU) was amplified using the primer pair ITS1OF (Taylor and McCormick, 2008) and TW14 (Bidartondo and Duckett, 2009). PCR products were cleaned using ExoSap-IT (Affymetrix). Clean PCR products were unidirectionally sequenced using the primer ITS1OF for ITS amplicons and LROR for LSU amplicons. Sanger sequencing was performed by either Genewiz (Cambridge, MA, USA) or ASGPB (University of Hawaiʻi, Honolulu, HI, USA).

### Fungal identification

Sequences were trimmed in Geneious (v.5.4.6). OTUs were binned at 97 % identity using the QIIME ‘open reference’ workflow (v1.9.1; Caporaso *et al.*, 2010). Conserved regions flanking ITS1 were removed using ITSx (v1.0.11; Bengtsson-Palme *et al.*, 2013). Open reference clustering was performed against the dynamic UNITE fungal database version 28.06.2017. Sumaclust was used during open reference OTU binning (Mercier *et al.*, 2013), and SortMeRNA (Kopylova *et al.*, 2016) was used for subsequent *de novo* OTU binning. OTU designation for sequences that contained ambiguous sites was confirmed by local alignment using MUSCLE (v. 3.8.31). Initial taxonomic assignments were based on Blast alignments with the non-redundant databases of NCBI and UNITE (Nilsson *et al.*, 2015). Queried sequences were considered a match at the generic level if the query coverage was 100 % and percent identity was ≥94 %, and a match at the familial or ordinal levels if the query coverage was ≥93 % or ≥83 %, and if percent identity was ≥90 % or ≥81 %, respectively (Table 1). Representative samples with ITS sequences matching fungi from known orchid mycorrhizal groups, specifically the family Ceratobasidiaceae or the genus *Ceratobasidium*, were resequenced with the LSU primer LROR for phylogenetic reconstruction. Representative ITS sequences for all OTUs and representative LSU sequences for Ceratobasidiaceae OTUs were deposited in GenBank (MF539822:MF539824, MF599203:MF599205 and MF574046:MF574052).

### Phylogenetic analyses

For phylogenetic reconstruction, ITS and LSU sequences were selected for each *Ceratobasidium* or Ceratobasidiaceae OTU based on sequence length and quality. Phylogenetic analyses were performed on two separate data sets. The first data set was comprised of concatenated ITS/LSU sequences representing each OTU and reference sequences of *Rhizoctonia* anamorphs in Ceratobasidiaceae from recently published phylogenies (Gónzalez *et al.*, 2001, 2016). Sequences of *Hydnum* and *Clavulina* were included as an outgroup. The second data set contained ITS sequences from each OTU, top Blast hits from NCBI’s GenBank and *Ceratobasidium* sequences from previous studies of orchid mycorrhizal fungi (Bidartondo *et al.*, 2004; Bougoure *et al.*, 2005; Girlanda *et al.*, 2005, 2011; Irwin *et al.*, 2007; Waterman and Bidartondo, 2008; Shefferson *et al.*, 2010; Sommer *et al.*, 2012; Nomura *et al.*, 2013). A closely related and putatively ectomycorrhizal sub-clade of *Ceratobasidium* was included as an outgroup (Tedersoo *et al.*, 2010; Veldre *et al.*, 2013). Alignments were performed using MUSCLE (Edgar, 2004) with the default settings. Alignments were visualized using Mesquite (v. 3.2). Regions too heterogeneous to align, and insertions/deletions that were present in less than half of the sequences were excluded from subsequent analyses. Aligned data sets were analysed using a maximum likelihood (ML) framework. Appropriate models of nucleotide substitution were selected for both data sets based on Akaike information criterion scores using the ‘automated model selection’ option implemented in *PAUP (v. 4.0a152, ^©^Swofford, 2016). For the ITS data set, the highest scoring model was HKY + I + G. For the concatenated data set, the highest scoring model was GTR + I + G. The HKY + I + G model is not implemented in RAxML so GTR + I + G was used as the model of nucleotide substitution for all analyses. For each data set, 100 independent ML searches were run in RAxML (Stamatakis, 2014) along with 2000 bootstrap replicates. Trees were visualized and annotated using the R program for Statistical Computing (v. 3.3.2; R Core Team, 2013) and the packages gg_tree v 1.6.11, ape v 4.1, dplyr v 0.5.0 and RColorBrewer v 1.1–2 (Paradis *et al.*, 2004; Neuwirth, 2014; Wickham and Francois, 2016; Yu *et al.*, 2017).

### Estimates of mycorrhizal specificity and distance decay relationship of mycorrhizal fungi among orchid populations

**Fig. 2.**
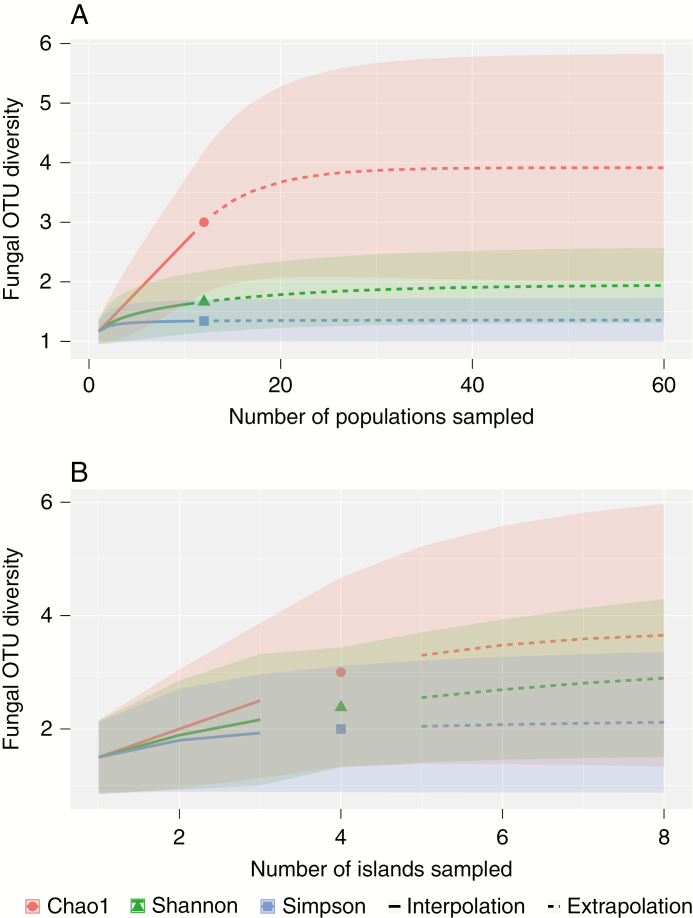
(A) Observed (symbols), interpolated (solid lines) and extrapolated (dashed lines) mycorrhizal fungal lineage richness and diversity from *Anoectochilus sandvicensis* populations based on the first three Hill numbers: Chao1 Richness, exponential Shannon diversity and reciprocal Simpson diversity; shaded areas represent the upper and lower 95 % confidence intervals for each estimator. (B) As (A), but calculated by island rather than orchid populations.

To assess whether our sampling efforts resulted in detection of the full range of fungi that associate with *A. sandvicensis*, we calculated the first three Hill numbers using the iNext package v 2.0.12 in R (Chao *et al.*, 2014). Because values of observed species richness often underestimates total richness (Hughes *et al.*, 2001), Hill numbers offer numerous advantages over reporting observed species richness alone as well as advantages over other richness estimators (Chao *et al.*, 2014). The first Hill number (*q* = 0) is simply an estimate of richness without regard for relative abundance. The second two Hill numbers (*q* = 1 and *q* = 2) are the exponential of Shannon entropy and the inverse Simpson concentration, respectively, which both incorporate species (or OTU) abundance into their estimates, but Shannon is the effective number of common OTUs whereas Simpson is the effective number of dominant OTUs in the assemblage. We used the number of sequences of each mycorrhizal OTU as a measure of taxon abundance. We estimated mycorrhizal richness and diversity both by *A. sandvicensis* population (Fig. 2A) and by island (Fig. 2B).

To test whether geographic proximity of *A. sandvicensis* populations was a significant predictor of mycorrhizal fungal community composition or the presence of particular *Ceratobasidium* OTUs, we performed Mantel correlation tests between geographic distance (in metres) and Bray–Curtis community dissimilarity for either all mycorrhizal taxa or only *Ceratobasidium* OTUs. Mantel test *P*-values were calculated based on 1000 permutations in the R package vegan version 2.4–6.

## RESULTS

The dominant fungal genus associated with populations of *Anoectochilus sandvicensis* was *Ceratobasidium*, where each orchid population partnered with a single OTU in this genus (Table 1). Overall, *Ceratobasidium* sequences accounted for 94 % of the total 237 sequences from this study, and in seven out of the 12 orchid populations sampled it was the only fungus detected. The additional fungi found in the remaining five populations included saprotrophs (two polypore OTUs represented by one and three sequences, respectively; two sequences of a single *Galerina* species, and four sequences of a single OTU in the order Trechisporales, Table 1). We also detected two additional putative mycorrhizal OTUs including the same Atheliaceae taxon (100 % query coverage and 99 % identity) found associating with non-native pines in plantations on Maui (Hynson *et al.*, 2013), here represented by three sequences, and a single Tulasnellaceae OTU represented by two sequences (Table 1). This Tulasnellaceae species was amplified with the ITS1OF/ITS4OF primers. None of our samples amplified fungi with the ITS1/ITS4-TUL primers. Overall, we had a PCR success rate of 87.9 % with the ITS1OF/ITS4OF primers.

Based on our binning of sequences at 97 % similarity, we detected three *Ceratobasidium* OTUs (Table 1). By far the most common was *Ceratobasidium* sp. 1, which was detected in eight populations of *A. sandvicensis* on three islands and represented by a total of 141 sequences (Table 1). *Ceratobasidium* sp. 2 was the next most common, found associating with three populations across two islands (Table 1) represented by 63 sequences, while *Ceratobasidium* sp. 3 was only detected from a single orchid population in the Wainiha Valley, Kauaʻi and represented by 18 sequences. Each of the 12 orchid populations partnered with a single OTU of *Ceratobasidium,* and all sites except Mt. Kaʻala on Oʻahu harboured only a single *Ceratobasidium* OTU (Table 1).

**Fig. 3.**
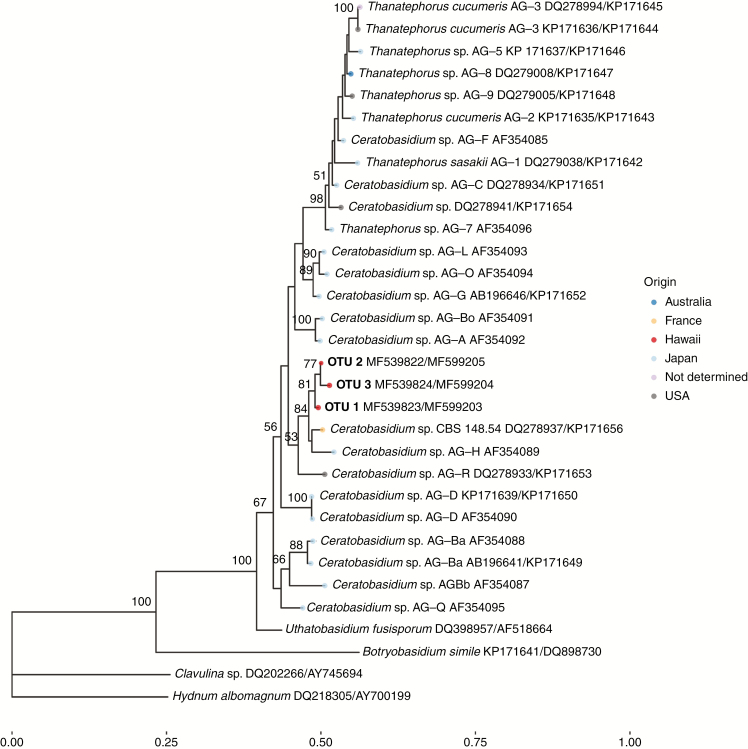
Phylogenetic placement of *Ceratobasidium* species isolated from *Anoectochilus sandvicensis*. Phylogenetic analysis was conducted on a combined ITS/LSU data set under a maximum likelihood framework as implemented in RAxML (Stamatakis, 2014). Branch support was assessed via 2000 bootstrap replicates. Bootstrap values >50 are shown. Representative taxa from the genera *Clavulina* and *Hydnum* were used as an outgroup for the analysis. The geographic origin of each sequence is indicated by colour.

The concatenated ITS + LSU phylogeny (Fig. 3) included expert curated reference sequences from *Ceratobasidium* anastomosis group AG and indicated that all three *Ceratobasidium* OTUs from our study formed a monophyletic clade with two *Ceratobasidium* OTUs in the AG-H and AG-R anastomosis groups from Japan and the USA, and another *Ceratobasidium* OTU (isolate CBS 148.54) from France (Fig. 3). Along with representative sequences from each of the three OTUs described in the current study, the concatenated data matrix included an additional 30 taxa from recent phylogenies of *Rhizoctonia* anamorphs within Ceratobasidiaceae (Gónzalez *et al.*, 2001, 2016). The final alignment contained 1755 characters of which 978 were included in the analysis. Of the included characters, 239 were parsimony informative.

**Fig. 4.**
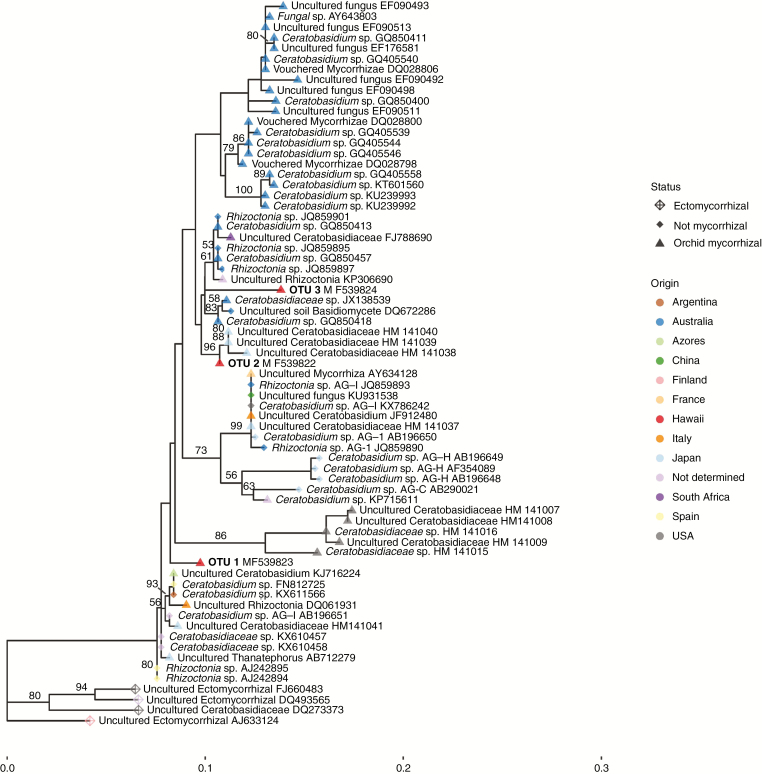
Phylogenetic placement of *Ceratobasidium* species isolated from *Anoectochilus sandvicensis* and highly similar (≤8.33 % pairwise sequence dissimilarity) mycorrhizal and non-mycorrhizal fungal sequences. The data set was comprised of ITS sequences and analysed under a maximum likelihood framework as implemented in RAxML (Stamatakis, 2014). Branch support was assessed via 2000 bootstrap replicates. Bootstrap values >50 are shown. The geographic origin of each sequence is indicated by colour, and the mycorrhizal status is indicated by shape.

The ITS phylogeny provided a finer scale phylogenetic context and was annotated with the geographic origin and mycorrhizal status of each sequence (Fig. 4). This data set included representative sequences from each of our three *Ceratobasidium* OTUs and 69 sequences of *Ceratobasidium* taxa from GenBank. The final alignment contained 1664 characters of which 533 were included in the analysis. Of the included characters, 105 were parsimony informative. The maximum pairwise distance among ITS sequences included in the analysis was 8.33 %. Based on our ITS phylogeny, the three *Ceratobasidium* OTUs described from *A. sandvicensis* did not form a monophyletic group (Fig. 4). *Ceratobasidium* sp. 2 formed a well-supported sub-clade with sequences of *Ceratobasidium* from a previous study of orchid mycorrhizal fungi in Japan (bootstrap support = 96; Shefferson *et al.*, 2010). *Ceratobasidium* spp. 1 and 3 were retained in the in-group but did not form well-supported sub-clades with each other or with any of the reference sequences included in the analysis (Fig. 3). The best supported clade that includes *Ceratobasidium* spp. 1 and 3 also includes *Ceratobasidium* sp. 2 as well as a suite of other taxa in Ceratobasidiaceae including both orchid mycorrhizal and non-mycorrhizal species, and were found in Argentina, Australia, the Azores archipelago, China, France, Italy, Japan, South Africa, Spain and the USA (Fig. 4).

Based on our calculations of the first three Hill numbers, we estimated that the total mycorrhizal fungal richness (*q* = 0) for all *A. sandvicensis* populations was 3.92 ± 1.96 (s.e.), whereas exponential Shannon’s diversity (*q* = 1) was 1.89 ± 0.38 and reciprocal Simpson’s diversity (*q* = 2) was 1.35 ± 0.21. These asymptotic values for estimated richness and diversity were reached by sampling a minimum of 11 populations (Fig. 2A). We found that total estimated richness (*q* = 0) for mycorrhizal fungi associated with *A. sandvicensis* by island was 3.75 ± 1.64, whereas exponential Shannon’s diversity (*q* = 1) was 3.05 ± 0.88 and reciprocal Simpson’s diversity (*q* = 2) was 2.13 ± 0.6. These asymptotic values for estimated richness and diversity were reached by sampling a minimum of two islands (Fig. 2B). Based on Mantel tests, we found no correlation between the geographic distances among populations of *A. sandvicensis* and the community composition of mycorrhizal fungi (*P* = 0.322), or the presence of particular *Ceratobasidium* OTUs (*P* = 0.344).

## DISCUSSION

The colonization of distant oceanic islands should favour species with the ability for long-distance dispersal (Carlquist, 1974). However, for symbiotic organisms, dispersal ability alone will not determine their geographic ranges. For horizontally transmitted symbioses to establish in new habitats such as islands, either the host and symbiont(s) must arrive within similar time frames (co-dispersal) or the host must encounter compatible symbionts that are already established (co-opting). Co-opting seems the most likely strategy for plants colonizing the most remote island locations such as Hawaiʻi, as it should theoretically increase chances of survival as long as a host is compatible with a wide range of local symbionts (i.e. a generalist). Conversely, if a host’s compatible symbiont(s) are few, but geographically widespread and abundant, this could also lead to successful island colonization. This latter scenario is likely to be the case for *A. sandvicensis*.

Contrary to our first hypothesis, we found that the obligate symbiotic orchid species *A. sandvicensis* is not a generalist, but that it partners with specific fungi in the genus *Ceratobasidium*. We circumscribed three fungal OTUs in this genus as the dominant associates of *A. sandvicensis*, and via phylogenetic reconstruction determined that they are closely related to taxa found throughout the globe (Table 1; Figs 3 and 4). Our most abundant and widespread *Ceratobasidium* taxon falls in a clade containing orchid mycorrhizal and non-mycorrhizal taxa from all the major continents except Antarctica, as well as an orchid mycorrhizal taxon from the Azores archipelago (Fig. 4). In addition to finding this taxon associating with *A. sandvicensis* populations across three distant and distinct islands that vary in age, size and habitats, we have also detected it in air samples from the Mauna Loa Observatory located on one of the highest peaks in the Hawaiian Islands, some 36 km from the closest *A. sandvicensis* (L. Tipton *et al*., unpubl. data). Interestingly, and counter to our second hypothesis, we found no statistically significant relationship between geographic proximity of *A. sandvicensis* populations and the similarity of their mycorrhizal communities or presence of particular *Ceratobasidium* OTUs. Combined, these results provide rare direct and indirect evidence of long-distance dispersal in fungi and insights into the ubiquity of *Certatobasidium* sp. 1 across the Hawaiian Islands.

Based on our estimates of fungal diversity, we found that populations of *A. sandvicensis* are predicted to harbour on average between one and four fungal taxa, while individual islands are estimated to have between two and four *A. sandvicensis* fungal associates present. Furthermore, our sampling efforts exceed the number of populations or islands necessary to reach community saturation for each of our estimators and have therefore probably identified the total diversity of fungi associated with *A. sandvicensis* (Fig. 2A, B). In the populations of *A. sandvicensis* where other potentially mycorrhizal lineages were detected (ex. Tulasnellaceae sp. from Oʻahu and Atheliaceae sp. from Kauaʻi, Table 1), they were in very low abundance and there is only prior evidence of Tulasnellaceae containing orchid mycorrhizal species (Dearnaley *et al.*, 2016). While we included these lineages in our estimates of fungal diversity associating with *A. sandvicensis*, our estimates of Simpson’s diversity (approx. 1 taxon per population and approx. 2 per island) are likely to be the most realistic, as this index is weighted by the dominant taxa (Fig. 2A, B). These findings indicate that the ability of *A. sandvicensis* to establish locally is at least in part limited by its high degree of mycorrhizal specificity. In light of the high degree of mycorrhizal specificity found in adult *A. sandvicensis* populations and the fact that many of Hawaiʻi’s endemic plant species, including orchids, face extinction, below-ground symbiotic partnerships should be taken into consideration in biological conservation and restoration efforts. Additional studies that test specificity at the germination stage in *A. sandvicensis*, which may be less than adult associations (Bidartondo and Read, 2008), as well as studies of the mycorrhizal associations of the other two endemic Hawaiian orchids are needed to understand native Hawaiian orchid recruitment in the context of their mycorrhizal associations.

Mycorrhizal specificity among continental endemics and more geographically widespread orchid species may occur due to factors different from those for island endemics such as *A. sandvicensis*. Based on classic Island Biogeography Theory, the species pool of fungal symbionts should be greater on continents than on highly isolated islands such as Hawaiʻi (MacArthur and Wilson, 2001). Therefore, the highly specific mycorrhizal associations of *A. sandvicensis* may be driven by the limited availability of compatible fungal symbionts rather than factors such as partner sanctions (Kiers *et al.*, 2011). Both Island Biogeographic Theory and Carlquist’s ideas regarding island colonization predict that abiotic filters (e.g. distance from regional species pools) shapes island biodiversity, but Carlquist (1974) expanded upon the basic theory of MacArthur and Wilson (2001) by adding biotic filters (e.g. symbiotic interactions) into his predictions. To develop both theories further, we suggest that the spatial distribution and abundance of symbionts in addition to island age, size and isolation should also be taken into consideration for predictions of island biodiversity.

Our results also offer new insights into tropical orchid mycorrhizal associations in general. Unlike other tropical orchid species, including relatives of *A. sandvicensis*, this species does not appear to be a mycorrhizal generalist-favouring Tulasnellaceae species, but forms highly specific partnerships with closely related taxa in the genus *Ceratobasidium*. Interestingly, Serendipitaceae spp. were absent from this study, providing additional support that they may be less common in the tropics (Jacquemyn *et al.*, 2017). The degree of mycorrhizal specificity we observed in *A. sandvicensis* is similar to that of the fully mycoheterotrophic orchid species *Corallorhiza maculata* and *C. mertensiana* where populations partner with single taxa of ectomycorrhizal fungi that are closely related (Taylor and Bruns, 1999). Based on the results of prior studies, there is evidence that *A. sandvicensis* is partially mycoheterotrophic (Hynson, 2016), and that differences in the degree of partial mycoheterotrophy among *A. sandvicensis* populations may be owed to differences in fungal partnerships (Hynson *et al.*, 2015). The current study also provides new evidence of a partially mycoheterotrophic species that forms relatively specialized mycorrhizal associations, which has previously only been found in a handful of other temperate ectomycorrhizal orchidaceous and ericaceous species (Taylor and Bruns, 1997, 1999; McCormick *et al.*, 2004; Girlanda *et al.*, 2005; Matsuda *et al.*, 2012; Hynson *et al.*, 2015).

### Conclusion

In summary, by examining an endemic tropical island species that is obligately symbiotic, new patterns have emerged. First, island colonization and microbial symbiont specificity are not mutually exclusive; secondly, symbiont abundance and distribution is likely a key factor in determining the distribution of their hosts; and thirdly, in the case of *A. sandvicensis* and possibly other island-inhabiting plant species, mycorrhizal specificity may be driven by a limited available pool of compatible fungal symbionts (i.e. island host plants are occupying only a portion of their fundamental niche). While fine-level symbiont specificity, such as that observed in *A. sandvicensis*, may somewhat limit host distributions, it is not surprising that an island endemic species partners with cosmopolitan symbionts. From the host’s perspective, this strategy makes sense, as it should increase host fitness over evolutionary time by increasing the probability that it will encounter a compatible symbiont. Here we highlight how new empirical data can challenge long-standing theories of island colonization, establishment and survival, at least in Hawaiʻi. In light of which, additional investigations of tropical island biogeography and biodiversity are desperately needed, especially for symbiotic organisms.
